# Expression of protein kinase A catalytic subunits in healthy and diseased mouse kidneys

**DOI:** 10.1007/s00424-026-03190-z

**Published:** 2026-06-26

**Authors:** Sally Fuchs, Michael Majer, Yuliang Ma, Manuela Harloff, Susan Taylor, Jens Schlossmann

**Affiliations:** 1 Department of Pharmacology, University of Regensburg, Regensburg, Germany; 2 Department of Pharmacology, University of California San Diego, San Diego, USA

**Keywords:** cAMP, Diabetic nephropathy, Diabetic kidney disease, Fibrosis, PKA, Protein kinase

## Abstract

Diabetic nephropathy (DN) is characterized by a decline in renal function resulting from hyperglycaemia and is often requiring dialysis or renal transplantation. Yet, the signalling events causing DN and the effective treatment options are poorly understood. Changes in the signalling of cyclic nucleotides and their regulated kinases are hypothesized to be involved in its development. Protein kinase A (PKA) signalling pathways are known to modulate extracellular matrix metabolism and exert antifibrotic effects. Multiple isoforms of PKA regulatory and catalytic subunits exist, leading to functional specificities of the kinase arising from different combinations of these isoforms. However, localization of the specific PKA subunits, as well as other signalling proteins involved in this pathway, still need to be explored comprehensively. To gain an overview about PKA distribution, kidneys were analysed by immunohistochemistry and stained for different PKA subunits. Type 1 diabetes was induced by streptozotocin in wildtype (WT) and endothelial NOS knockout (eNOS-KO) mice. The catalytic subunit expression was quantified and compared between healthy and diabetic kidneys. Analysis of expression patterns of the PKA catalytic subunits Cα and Cβ reveal differences across segments of the kidney and in intracellular localization. Cα exhibited ubiquitous expression in all renal cell types. In contrast, Cβ only shows a high expression in proximal tubules, while its expression in other segments is comparatively weak. No significant changes in Cα or Cβ expression are detectable in diabetic mice or eNOS-KO mice compared to WT mice.

**Supplementary Information:**

The online version contains supplementary material available at 10.1007/s00424-026-03190-z.

## Introduction

Diabetic kidney disease, also known as diabetic nephropathy (DN), is a major complication of a poorly adjusted diabetes mellitus. It is characterized by an expansion of mesangial matrix proteins and glomerulosclerosis, leading to a decline in filtration rate. Furthermore, the metabolic changes cause tubulointerstitial inflammation and fibrosis [1, 2]. Despite recent advances, treatment options for diabetic kidney disease remain limited. Hence, it is necessary to understand the underlying molecular mechanisms involved in these pathologies.

The second messenger cyclic adenosine monophosphate (cAMP) is an important signalling molecule regulating different kidney functions by activation of the cAMP-dependent protein kinase, also called protein kinase A (PKA). Alterations in the signalling of this cyclic nucleotide and its regulated kinase contribute to the development of various chronic kidney diseases (CKDs), including fibrosis, diabetic nephropathy, and polycystic kidney disease (PKD) [3, 4]. PKA was firstly described in 1968 by Walsh et al. [5]. It is a tetrameric holoenzyme consisting of a regulatory subunit dimer and two catalytic subunits (PKAc). Mammalian cells possess four regulatory subunit isoforms (RIα, RIβ, RIIα and RIIβ) and three catalytic subunit isoforms (Cα, Cβ and Cγ) of PKA. In its inactive state, each regulatory subunit binds a catalytic subunit to supress its activity. The regulatory subunits contain the cyclic nucleotide-binding (CNB) domains that function as a receptor for cAMP. Intracellular levels of cAMP in cells are controlled by production from adenylyl cyclase (AC) and degradation by phosphodiesterases (PDEs). Upon binding of cAMP to the regulatory subunits and activation of the kinase, the catalytic subunits dissociate from the regulatory subunits and are now able to phosphorylate their target proteins. Importantly, the specificity of PKA signalling is tightly regulated by cell type-specific isoform expression, subcellular localization, and substrate availability [6]. Spatial control of PKA activity is achieved through interactions with A-kinase anchoring proteins (AKAPs), which localizes PKA to specific subcellular compartments [7, 8]. Thus, PKA can function in the cytoplasm as well as in the nucleus, where it regulates gene transcription by phosphorylation of transcription factors like the cAMP-responsive element binding protein (CREB) [6].

**Fig. 1:**
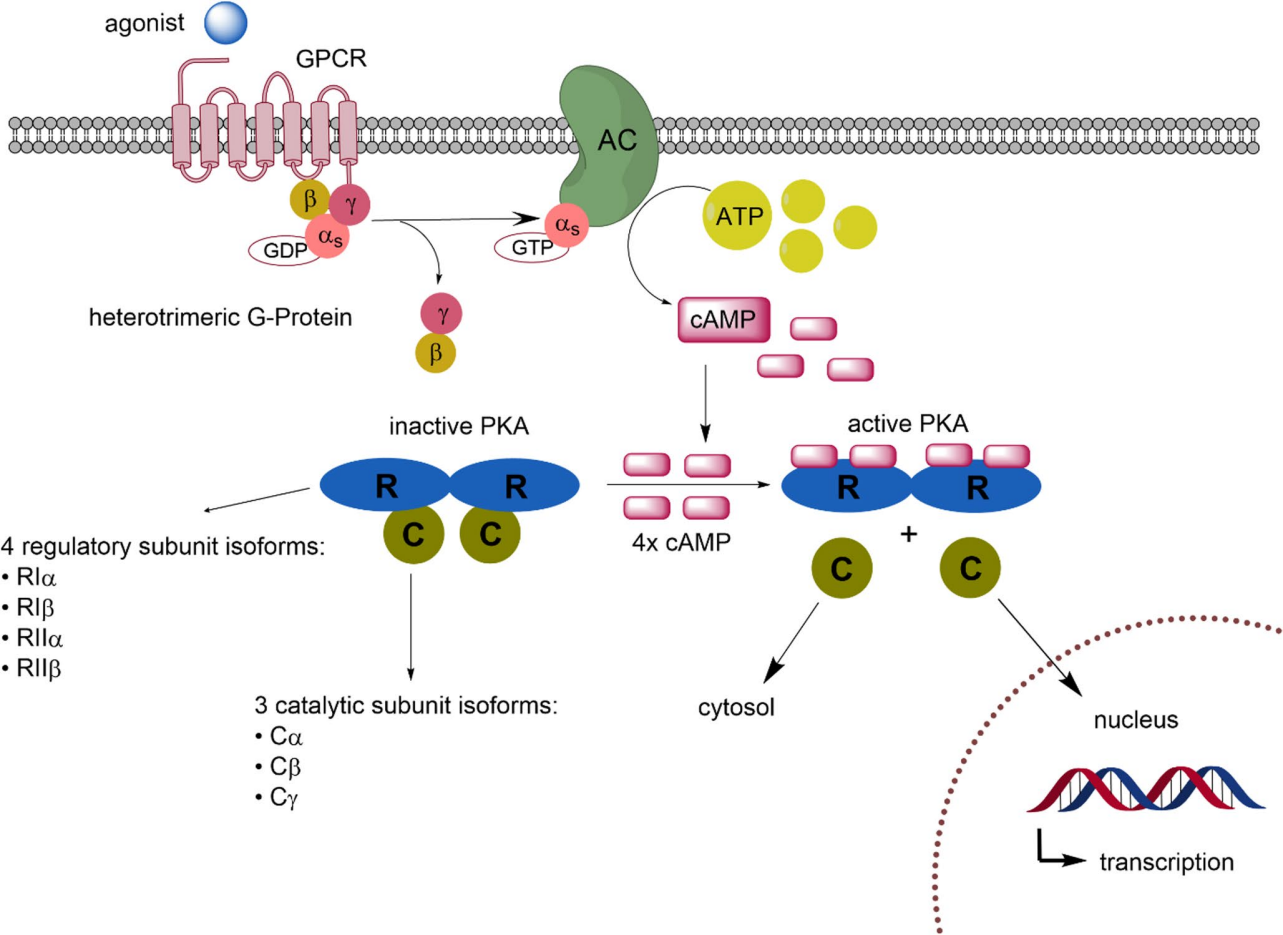
cAMP/PKA signalling in mammalian cells. After Gαs-mediated activation of adenylyl cyclase (AC) by stimulation of a G-Protein coupled receptor (GPCR), cAMP is generated, which activates PKA by binding to its regulatory subunits. In its inactive state, two regulatory subunits are bound as a dimer to two catalytic subunits. Upon activation through cAMP, the catalytic subunit is released from its regulatory subunit and phosphorylates target proteins in the nucleus or the cytosol. Mammalian cells express three different catalytic subunit isoforms (Cα, Cβ, Cγ) and four different regulatory subunit isoforms (RIα, RIβ, RIIα, RIIβ). This image was created using ChemDraw version 23.0.1

The PKA signalling pathway (Fig. 1) regulates several biological and physiological events. Mutations of PKA subunits, as well as aberrant or dysfunctional PKA signalling, are linked to many diseases, including neurodegenerative, cardiovascular, and metabolic disorders, to name only a few [9–11]. In the kidney, recent efforts have demonstrated that downregulation of PKA delays the development and progression of PKD. A novel therapeutic strategy involves the use of a small molecule that inhibits Cα activity, which has been validated in a mouse model of polycystic kidney disease [4]. Furthermore, PKA signalling exerts antifibrotic effects, highlighting the pathway a potential target for treatment of kidney fibrosis, a key pathological feature associated with DN [3]. The given examples demonstrate that PKA signalling plays a substantial role in kidney pathology. However, considering PKA as a potential drug target for kidney diseases requires a complete understanding of its different isoforms and their involvement in the cellular mechanisms of kidney disease, which remains poorly understood despite high therapeutic potential. Thus, we critically aimed to investigate the expression of these isoforms in the different cell types of the kidney to elucidate specific functions within each segment. In our study, we provide a complete overview on the expression of PKA catalytic subunits in the different segments of the kidney with focus on the isoforms Cα and Cβ. In addition, we are investigating their expression in fibrotic tissue of diabetic animals. To this end, we are using a mouse model in which type 1 diabetes is induced in wild-type (WT) and endothelial NOS knockout (eNOS-KO) mice over a period of 12 weeks by streptozotocin. The eNOS-KO mice, in particular, develop advanced diabetic nephropathy that exhibit many features of the human disease [12]. Furthermore, especially in diabetic eNOS-KO mice a strong increase of serum creatinine and a significant decline in GFR are detectable, indicating progressive renal dysfunction [13]. Our study demonstrates that there are differences in expression of Cα and Cβ in the fibrotic tissue of diabetic animals.

## Materials and methods

### Animals

All animal care and experimental procedures were performed in accordance with the Guidelines for Care and Use of Laboratory Animals published by the US National Institutes of Health and were approved by the local authorities (Regierung von Unterfranken, RUF-55.2-2532-2-400). eNOS knockout mice (eNOS-KO; B6.129P2-*Nos3*^*tm1Unc*^/J) were obtained from Jackson Laboratories (USA). Wildtype (WT) and eNOS-KO mice (both with Sv129/C57BL/6J background) were bred and maintained under pathogen-free conditions in the animal facilities of University of Regensburg with free access to standard rodent chow and tap water and were housed in a 12:12 h light–dark cycle under controlled temperature conditions.

### Induction of type 1 diabetes

Type 1 diabetes mellitus (DM) was induced in 8- to 12-week-old male WT or eNOS-KO mice by i.p. injection of streptozotocin (STZ) (50 mg×kg^− 1^×day^− 1^, freshly dissolved in 50 mM sodium citrate buffer pH 4.5) on five consecutive days as previously described [13, 14]. During the treatment with STZ, 10% sucrose was added to the drinking water. Ten days after the last injection, manifestation of diabetes mellitus was verified by measuring the fasting blood glucose from tail blood using a glucose meter (Contour^®^XT, Bayer, Germany). Mice with glucose levels ≥ 15.5 mmol×L^− 1^ were classified as diabetic and were allowed to develop DN for further 12 weeks. Diabetic eNOS-KO mice developed pathological features similar to human DN [12, 15]. The extent of diabetic nephropathy in these animals was already analysed before by Harloff et al. [13].

### Immunohistochemistry

Animals were perfuse-fixed with 0.9% NaCl followed by 3% PFA. Afterwards, kidneys were excised and stored in 70% methanol prior to paraffin embedding according to previous protocols [16]. For immunohistochemistry, 2.5 μm sections of paraffin embedded kidneys were prepared using a microtome (Thermo Scientific, Germany). Slices were dewaxed, rehydrated (2 × 10 min xylene, 3 × 5 min isopropanol, 2 × 5 min 100% methanol, 2 min ddH_2_O) and blocked with 10% horse serum in 1% BSA for 2 h. Afterwards, slides were incubated with primary antibodies overnight at 4 °C, followed by incubation with respective fluorophore-coupled secondary antibody at room temperature for 2 h. Nuclear counterstaining was performed using DAPI. After washing, slides were mounted with Dako Glycergel Mounting Medium (Agilent, USA). Details of the used primary and secondary antibodies are listed in Table 1. Fluorescence was detected with a Zeiss Axiovert 200 microscope (Zeiss, Germany). Confocal images were captured using a Zeiss LSM 980 Confocal microscope with Airyscan (Zeiss, Germany) and a Nikon Eclipse Ti2-E Laser Scanning A1R-Confocal microscope (Nikon, USA). Intensity of fluorescence was assessed using the ImageJ (Fiji, National Institute of Health, USA) software. Sections incubated only with secondary antibodies were used as negative controls and are shown in supplementary figures (Online Resource 1, 2 and 3c).

**Table 1:** Primary and secondary antibodies used for immunohistochemistry

Antibody	Species	Manufacturer		Dilution
Anti PKA-Cα	rabbit	production laboratory Susan Taylor		1:100
Anti PKA-Cβ	rabbit	Abcepta, #AP51447		1:100
Anti PKA-RIα	mouse	BD-Transduction Laboratories, #610,609		1:10
Anti PKA-RIβ	sheep	R&D Systems, #AF4177		1:300
Anti PKA-RIIα	mouse	Santa Cruz, #sc-137,220		1:100
Anti PKA-RIIβ	mouse	BD-Transduction Laboratories, #610,625		1:100
Anti α8-integrin	goat	R&D Systems, #AF4076		1:100
Anti AQP2	goat	Santa Cruz, #sc-9882		1:200
Anti calbindin	mouse	Swant, #D28K		1:500
Anti CD31	goat	R&D Systems, #AF3628		1:25
Anti megalin	mouse	Santa Cruz, #sc-515,772		1:200
Anti renin	goat	R&D Systems, #AF4277		1:50
Anti NKCC2	mouse	Santa Cruz, #sc-293,222		1:50
Anti α-SMA	mouse	Abcam, #ab7817		1:600
Anti synaptopodin	goat	Santa Cruz, #sc-21,537		1:200
Alexa 488 donkey anti rabbit	donkey	Invitrogen, #A21206		1:250
Alexa 488 donkey anti mouse	donkey	Invitrogen, #A21202		1:250
Alexa 546 donkey anti goat	donkey	Invitrogen, #A110566		1:250
Alexa 546 donkey anti mouse	donkey	Invitrogen, #A10036		1:250
Alexa 647 donkey anti mouse	donkey	Invitrogen, #A31571		1:250
Cy3 donkey anti sheep	donkey	Jackson Lab, #713-165-003		1:100

### Statistics

Statistical analysis was performed using GraphPad Prism (version 11.0.2, GraphPad software Inc., USA) with each group size *n* = 5. The Shapiro Wilk test was used to test for normal distribution. Normally distributed parameters were analysed using a one-way ANOVA followed by Bonferroni’s post hoc tests. The post hoc tests were conducted only if *F* in ANOVA achieved *p* < 0.05 and there was no significant variance in homogeneity. Not normally distributed parameters were analysed using the Kruskal-Wallis test followed by Dunn’s multiple comparisons test. Data are presented as mean ± SEM. Adjusted *p*-values from the respective post hoc tests are displayed in the graphs. All data analyses were performed by an investigator who was blinded to the experimental group allocation until completion of the primary statistical analyses.

## Results

### Expression of different PKA catalytic and regulatory subunits in healthy murine kidneys

**Fig. 2:**
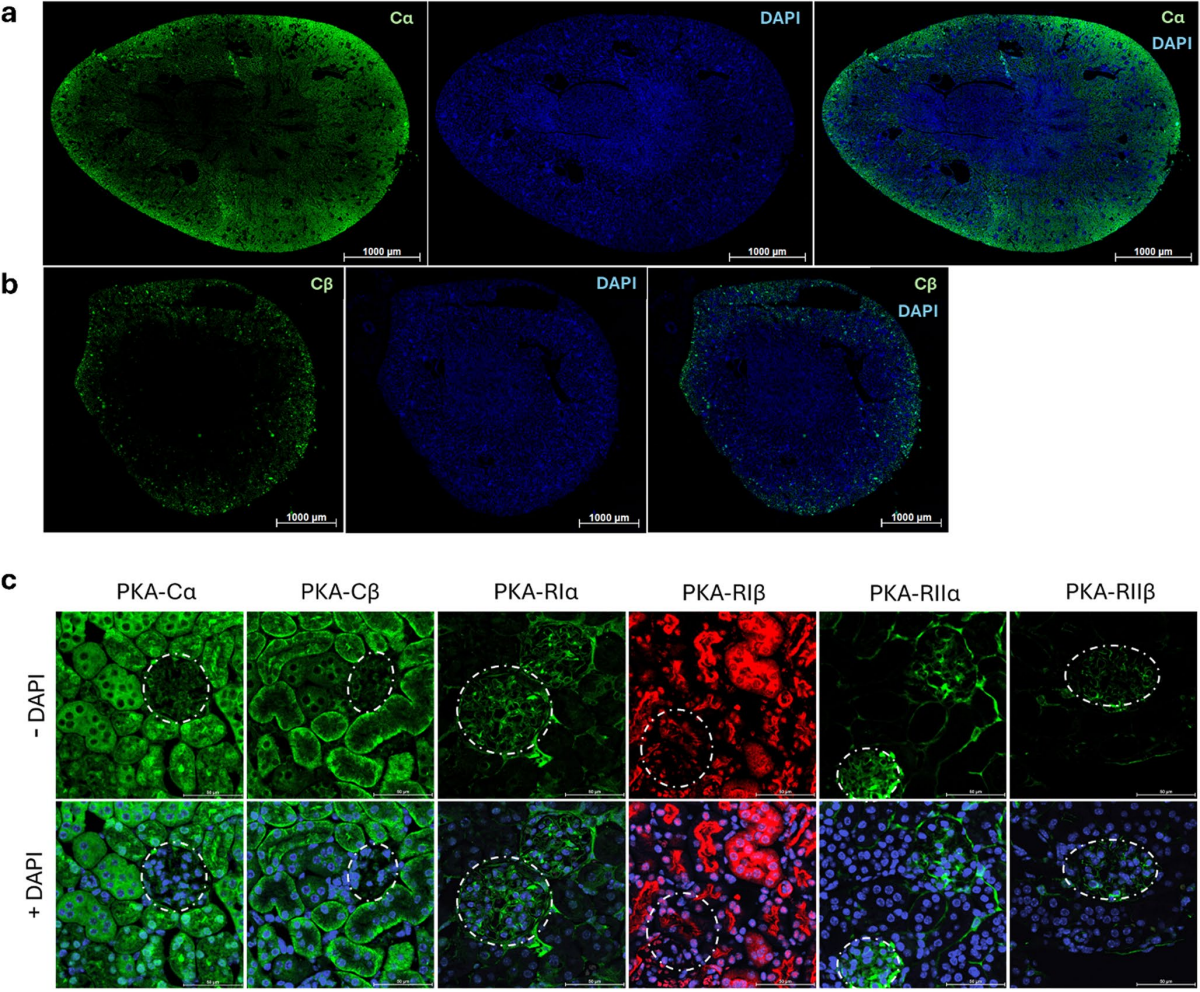
Overview about expression of Cα (**a**) and Cβ (**b**) in murine kidneys and expression of different PKA regulatory and catalytic subunit isoforms in the renal cortex (**c**). **a** - **b** Cα (**a**) and Cβ (**b**) are mainly expressed in the renal cortex. Cα (**a**) is strongly expressed in various cell types of the renal cortex, whereas Cβ (**b**) appears to be expressed only in a few specific cell types. **c** The regulatory und catalytic subunit isoforms are differently localized in the in the renal cortex. Glomeruli are visualized by white dashed lines. Nuclei are stained with DAPI (blue). Scale bar is 1000 μm (**a**, **b**) and 50 μm (**c**)

First, expression of the PKA catalytic isoforms Cα and Cβ was analysed in healthy, murine kidneys. Overview images show that Cα is broadly expressed throughout the kidney, with a strong expression in the cortex (Fig. 2a) and a lower amount of expression in the medulla, which were detectable at higher magnification (Online Resource 3a). We detected the Cβ isoform was predominantly localized to the cortex, with high expression limited to certain cell types (Fig. 2b). In the medulla, only a very weak expression was detectable when taking higher magnification images (Online Resource 3b). Precise profiling of the expression pattern of Cα and Cβ in the renal cortex reveals that Cα is expressed in every cell type of the renal cortex. Interestingly, in some cells, Cα is distributed over the whole cell body but not in the nucleus, while other cells show a more pronounced expression in the nuclei (Fig. 2c). Cβ, however, is rather localized to the basolateral membrane of specific cells as opposed to the nuclei (Fig. 2c). As both catalytic subunits are predominantly expressed in the cortex, we also analysed expression of the regulatory subunits in the renal cortex. We detected RIα, RIIα and RIIβ in the glomeruli. RIβ is also weakly expressed in glomeruli, where it is localized to the nuclei. Furthermore, a high expression of RIβ is detectable in the tubular system, where some cells show a high expression of RIβ distributed over the cytosol. Other cells, however, express RIβ rather at their apical membrane as well as in their nuclei (Fig. 2c).

### Localization of Cα and Cβ in healthy murine kidneys

To further distinguish which specific cells express Cα and Cβ and identify their subcellular localization in the different cell types, either Cα or Cβ were co-stained with specific markers for each cell type (Table 2).

**Table 2:** Markers for renal cell types

marker	cell type
CD31	glomerular capillaries
synaptopodin	podocytes
α8-integrin	mesangial cells
megalin	proximal tubules
NKCC2	henle loop
calbindin	distal tubules
AQP2	collecting ducts
renin	juxtaglomerular cells

### Localization of Cα and Cβ in glomeruli

**Fig. 3:**
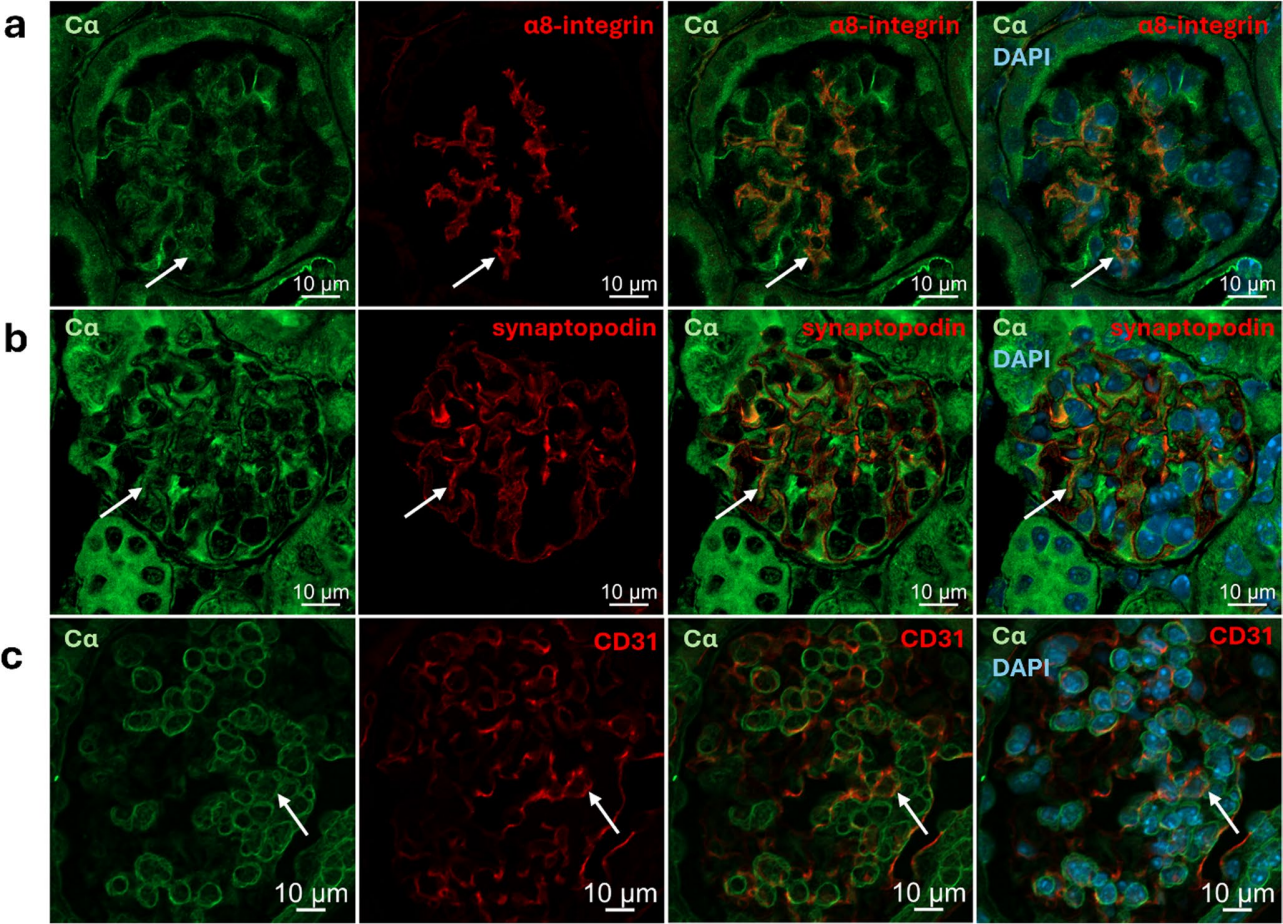
Expression of Cα in glomerular cells. **a** Costaining of Cα (green) and α8-integrin (red) indicates expression of Cα in mesangial cells. **b** Costaining of Cα (green) and synaptopodin (red) shows expression of Cα in podocytes. **c** Costaining of Cα (green) and CD31 (red) reveals expression of Cα in glomerular capillaries. Nuclei are stained with DAPI (blue). Expression in the cell type is indicated by arrows. Images are taken on a confocal microscope and scale bars are 10 μm

**Fig. 4:**
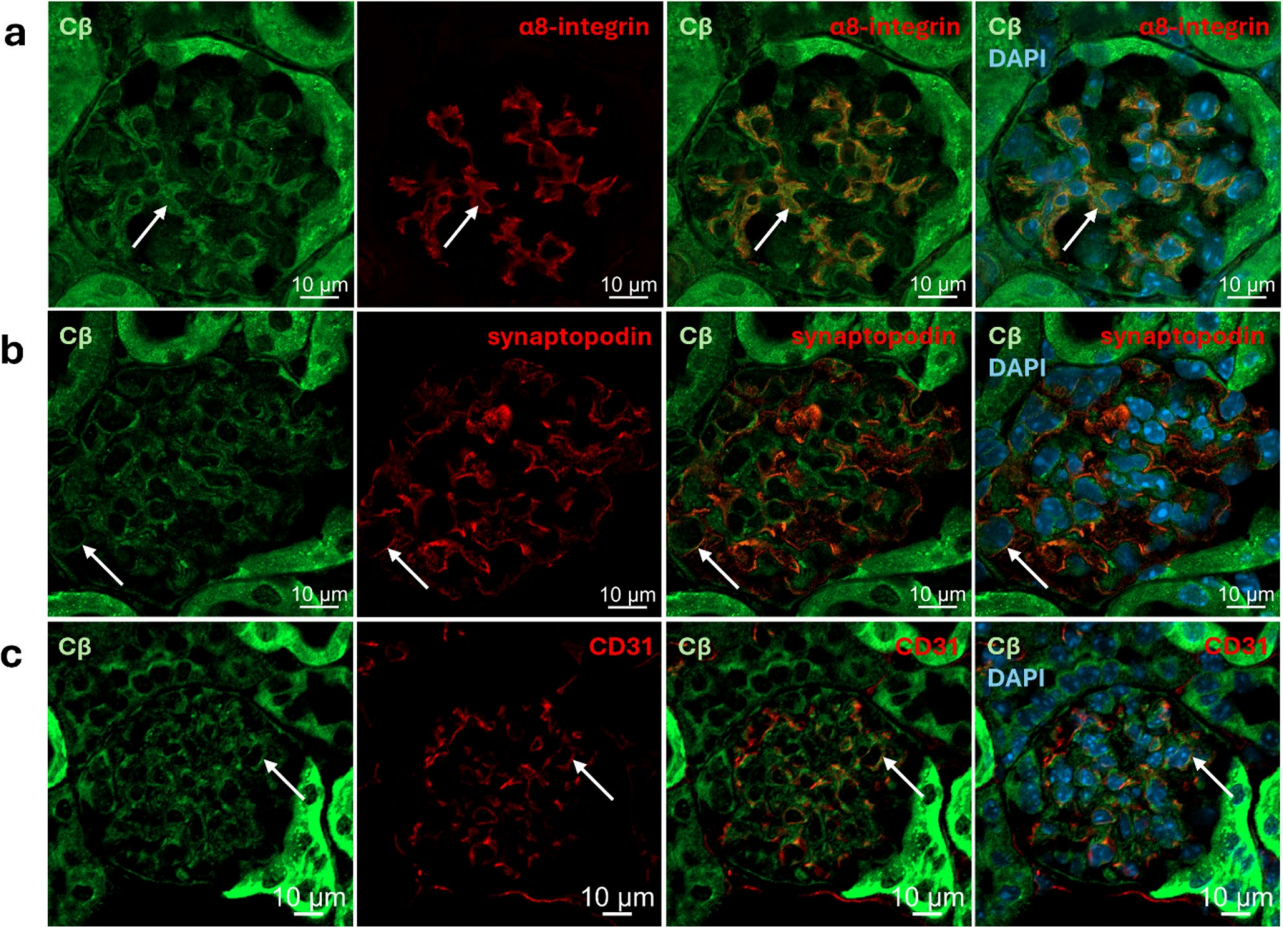
Expression of Cβ in glomerular cells. **a** Costaining of Cβ (green) and α8-integrin (red) indicates expression of Cβ in mesangial cells. **b** Costaining of Cβ (green) and synaptopodin (red) shows expression of Cβ in podocytes. **c** Costaining of Cβ (green) and CD31 (red) reveals expression of Cβ in glomerular capillaries. Nuclei are stained with DAPI (blue). Expression in the cell type is indicated by arrows. Images are taken on a confocal microscope and scale bars are 10 μm

The glomerulus is the renal structure responsible for filtering blood plasma and selectively allowing substances to pass through the glomerular filter. Important parts of the glomerulus include the glomerular capillaries, podocytes as part of the filtration barrier, and the glomerular mesangium with its mesangial cells. For localization analysis of Cα and Cβ inside the glomerulus, we costained either Cα or Cβ with the markers α8-integrin (mesangial cells), synaptopodin (podocytes) or CD31 (glomerular capillaries). Both Cα (Fig. 3a-c) and Cβ (Fig. 4a-c) were detectable in mesangial cells, podocytes and glomerular capillaries. However, neither Cα nor Cβ was detectable in the nuclei of these cells.

### Localization of Cα and Cβ in the tubular system

**Fig. 5:**
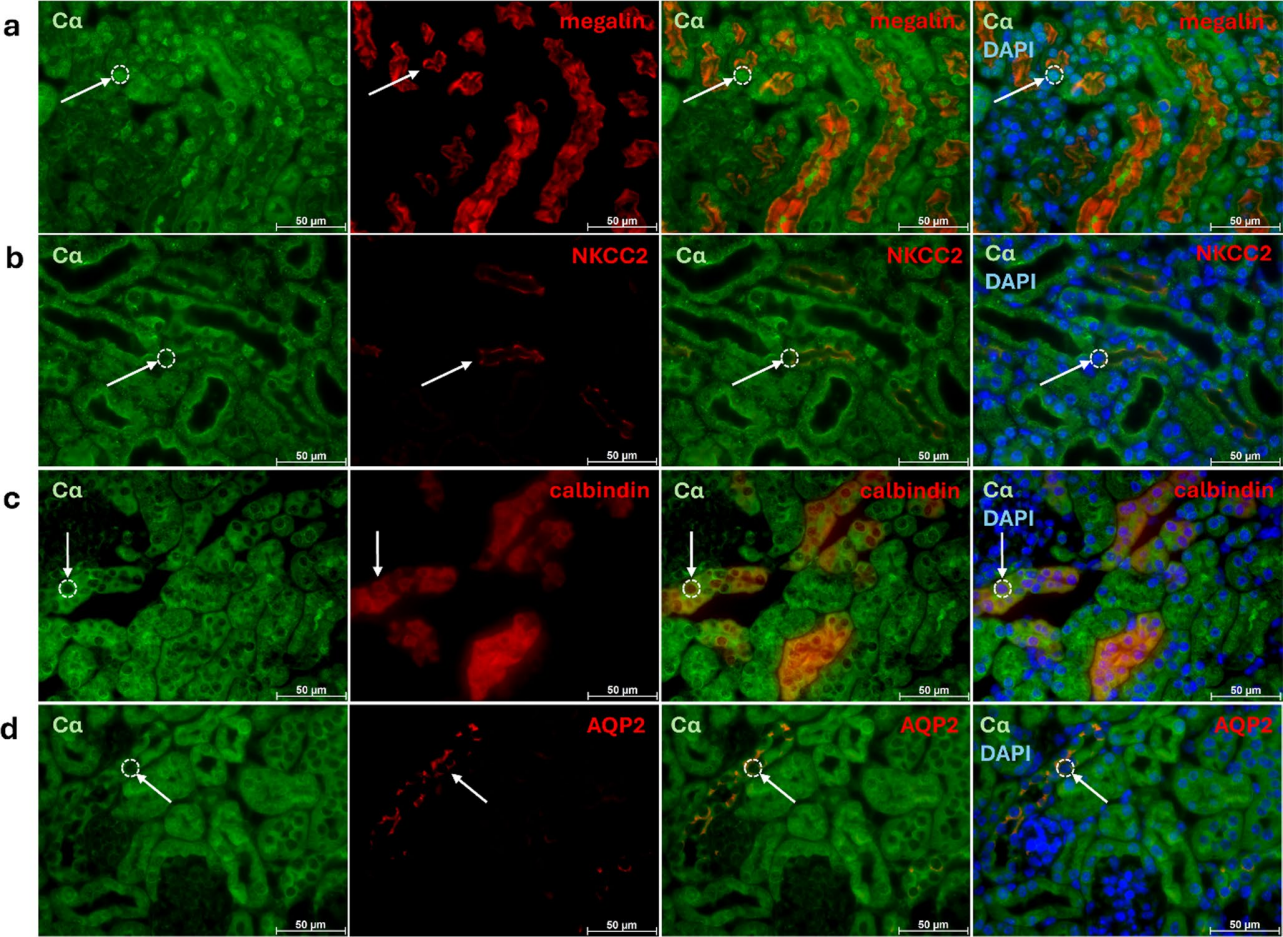
Expression of Cα in the tubular system. **a** Costaining of Cα (green) and megalin (red) shows expression of Cα in proximal tubules. In the proximal tubule cells Cα is strongly expressed in the nuclei. **b** Costaining of Cα (green) and NKCC2 (red) reveals expression of Cα in the Henle loop, where Cα is not detectable in the nuclei. **c** Costaining of Cα (green) and calbindin (red) demonstrates expression of Cα in distal tubules with no expression in the nuclei. **d** Costaining of Cα (green) and AQP2 (red) shows expression of Cα in collecting ducts. Cα is not detectable in the nuclei of these cells. Nuclei are stained with DAPI (blue) and are visualized by white dashed lines. Expression in the cell type is indicated by arrows. Scale bars are 50 μm

After blood is filtered in the glomerulus, the produced primary urine is collected in the Bowman’s capsule, followed by an extensive modification of the primary urine during its passage through the tubular system. Cα or Cβ were analysed for its expression in the different segments of the tubular system to evaluate possible functions in these segments. We analysed expression in proximal tubules (megalin), the Loop of Henle (Na^+^/K^+^/2Cl^−^-cotransporter; NKCC2), distal tubules (calbindin) and collecting ducts (aquaporin 2; AQP2). Costaining of Cα with megalin shows expression of Cα in proximal tubules (Fig. 5a). Interestingly, Cα is not only distributed over the whole cytosol of the proximal tubule cell but is also strongly expressed in their nuclei (Figs. 5a and 7a).

Furthermore, Cα expression is detectable in the Loop of Henle (Fig. 5b), distal tubules (Fig. 5c) and collecting ducts (Fig. 5d). In these cells, however, Cα shows only a cytoplasmic expression with no detectable nuclear expression (Fig. 5b-d).

**Fig. 6:**
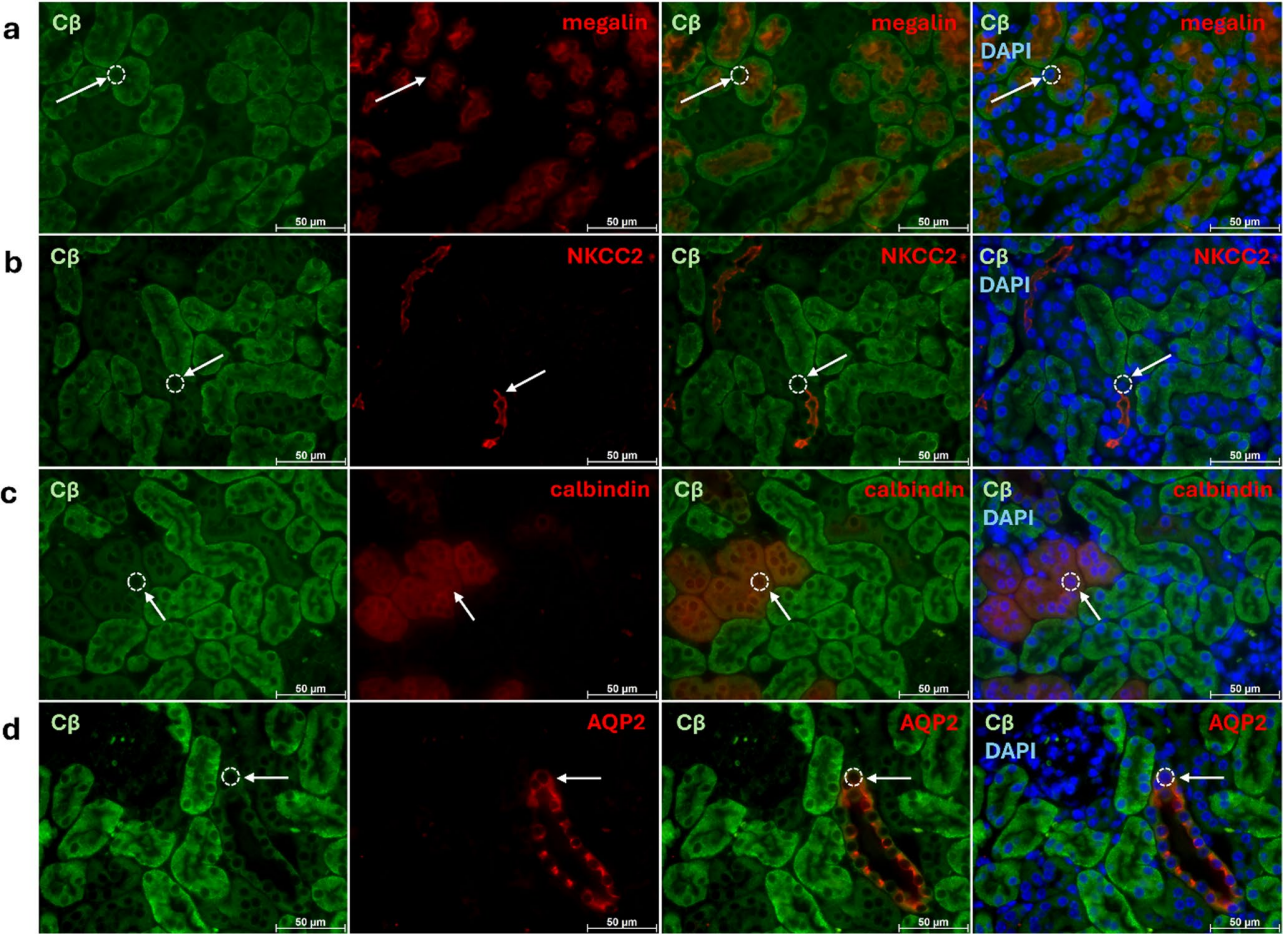
Expression of Cβ in the tubular system. **a **Costaining of Cβ (green) and megalin (red) shows strong expression of Cβ in proximal tubules. **b **Costaining of Cβ (green) and NKCC2 (red) reveals weak expression of Cβ in the Henle loop. **c **Costaining of Cβ (green) and calbindin (red) shows only low expression of Cβ in distal tubules. **d **Costaining of Cβ (green) and AQP2 (red) indicates low expression of Cβ in collecting ducts. **a-d** Cβ is not detectable in the nuclei of these cells. Nuclei are stained with DAPI (blue) and are visualized by white dashed lines. Expression in the cell type is indicated by arrows. Scale bars are 50 μm

**Fig. 7:**
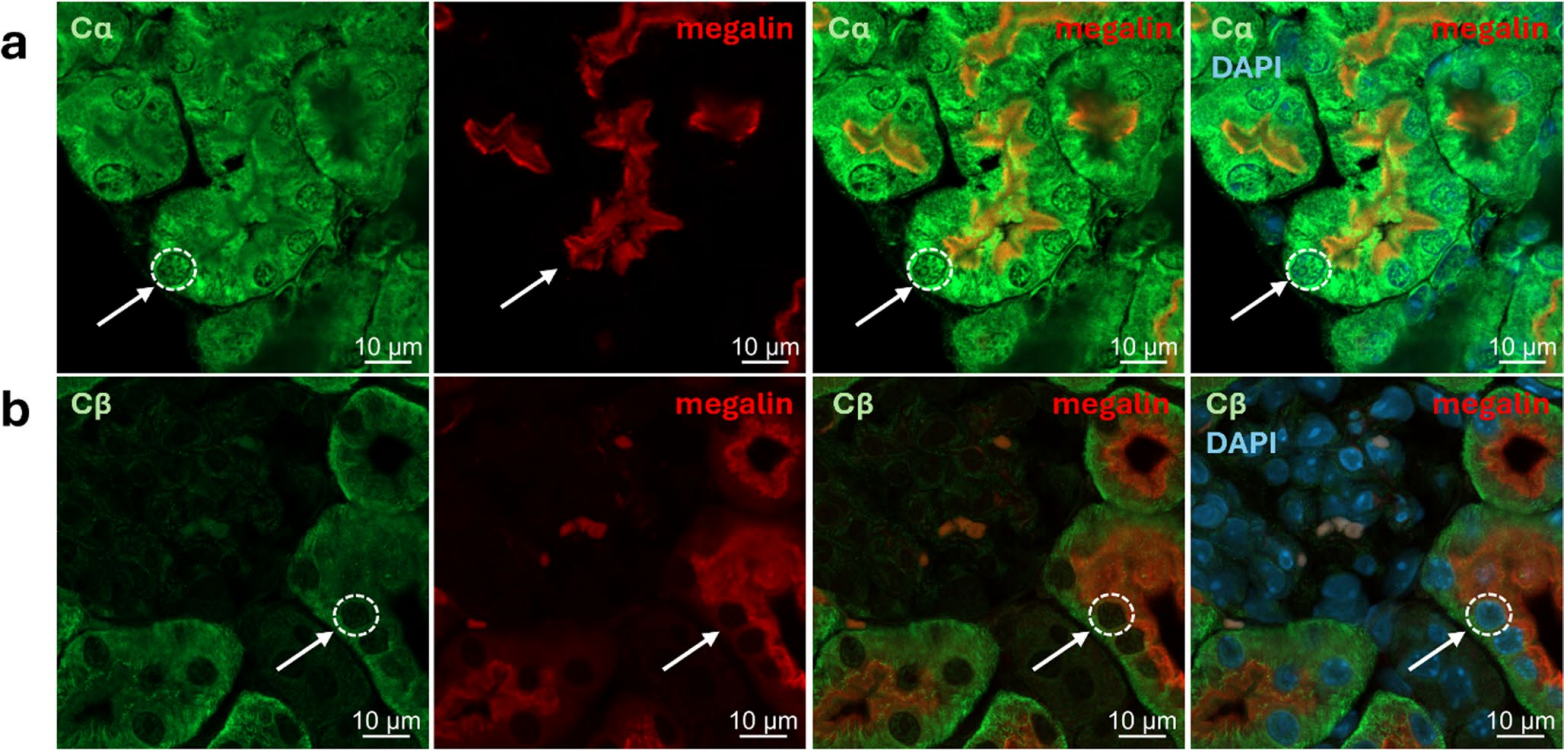
Expression of Cα and Cβ in proximal tubules. Confocal images of Cα and Cβ (green) show a high amount of Cα (**a**) and Cβ (**b**) in the proximal tubules (stained with megalin, red). Cα is distributed overthe whole cell and is also expressed in the nuclei (**a**), whereas Cβ shows its strongest expression at thebasolateral membrane but is not expressed in the nuclei (**b**). Nuclei are stained with DAPI (blue) and are visualized by white dashed lines. Expression in the cell type is indicated by arrows. Scale bars are 10 μm

Cβ shows strongest expression in proximal tubules (Fig. 6a), where it appears to be localized especially at the basolateral membrane of the cell (Figs. 6a and 7b). However, only weak expression of Cβ is detectable in the Loop of Henle (Fig. 6b), distal tubules (Fig. 6c) or collecting ducts (Fig. 6d). Furthermore, we did not observe expression of Cβ in the nuclei of the respective cell types (Fig. 6a-d).

As both Cα and Cβ revealed highest expression in the tubular system and were highly expressed in proximal tubules where their intracellular localization differed, we analysed which regulatory PKA subunit is co-expressed in the proximal tubules. As we already detected RIβ expression in the tubular system (Fig. 2c) we costained Cα or Cβ with RIβ. Costaining of Cα and RIβ reveal a colocalization of Cα and RIβ in proximal tubule cells (Online Resource 4a, b). Proximal tubule cells were identified as this was the only cell type where Cα was detectable in the nucleus. Interestingly, like Cα, RIβ is also expressed in the nuclei of the proximal tubule cells. Furthermore, expression of RIβ was detectable at the apical membrane of proximal tubules (Online Resource 4a, b). Costaining of Cβ and RIβ also revealed a colocalization of Cβ and RIβ in proximal tubule cells (Online Resource 5a, b). While Cβ is rather located at the basolateral membrane, RIβ is more distributed to the apical membrane (Online Resource 5a, b).

### Localization of Cα and Cβ in juxtaglomerular cells

**Fig. 8:**
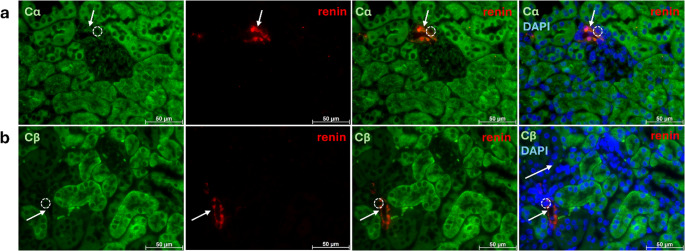
Expression of Cα and Cβ in juxtaglomerular cells. Expression of Cα (**a**) and only very weak expression of Cβ (**b**) is observed in juxtaglomerular cells, which are stained with renin (red). Neither Cα (**a**) nor Cβ (**b**) are detectable in the nuclei of the juxtaglomerular cells (**a**). Nuclei are stained with DAPI (blue) and are visualized by white dashed lines. Expression in the cell type is indicated by arrows. Scale bars are 50 μm

Juxtaglomerular cells are part of the juxtaglomerular apparatus and are localized at the vascular pole of the glomerulus. To investigate the expression of Cα or Cβ in the juxtaglomerular cells, kidney sections were costained with antibodies against renin and either Cα or Cβ. Expression of Cα was detectable in juxtaglomerular cells, with no expression of Cα in the nuclei (Fig. 8a). However, we saw only a very weak expression of Cβ in juxtaglomerular cells. Furthermore, no expression of Cβ was detectable in their nuclei (Fig. 8b).

### Localization of Cα and Cβ in fibrotic tissue of diabetic kidneys

Type 1 diabetes was induced in mice by using streptozotocin. In addition to WT mice, eNOS-KO mice were used, which lack endothelial NO-synthase and therefore reveal a more severe type of diabetes and renal fibrosis [12, 13]. We have previously investigated the extent of diabetic nephropathy in these mice and were able to show that induction of diabetes by STZ in WT and eNOS-KO mice leads to significantly elevated blood glucose levels and reduced body weight. Diabetic WT and diabetic eNOS-KO animals exhibit glomerular hypertrophy, higher urinary protein content and albuminuria, mesangial expansion and enhanced production and deposition of extracellular matrix proteins. However, these outcomes are more pronounced in diabetic eNOS-KO mice [13].

**Fig. 9:**
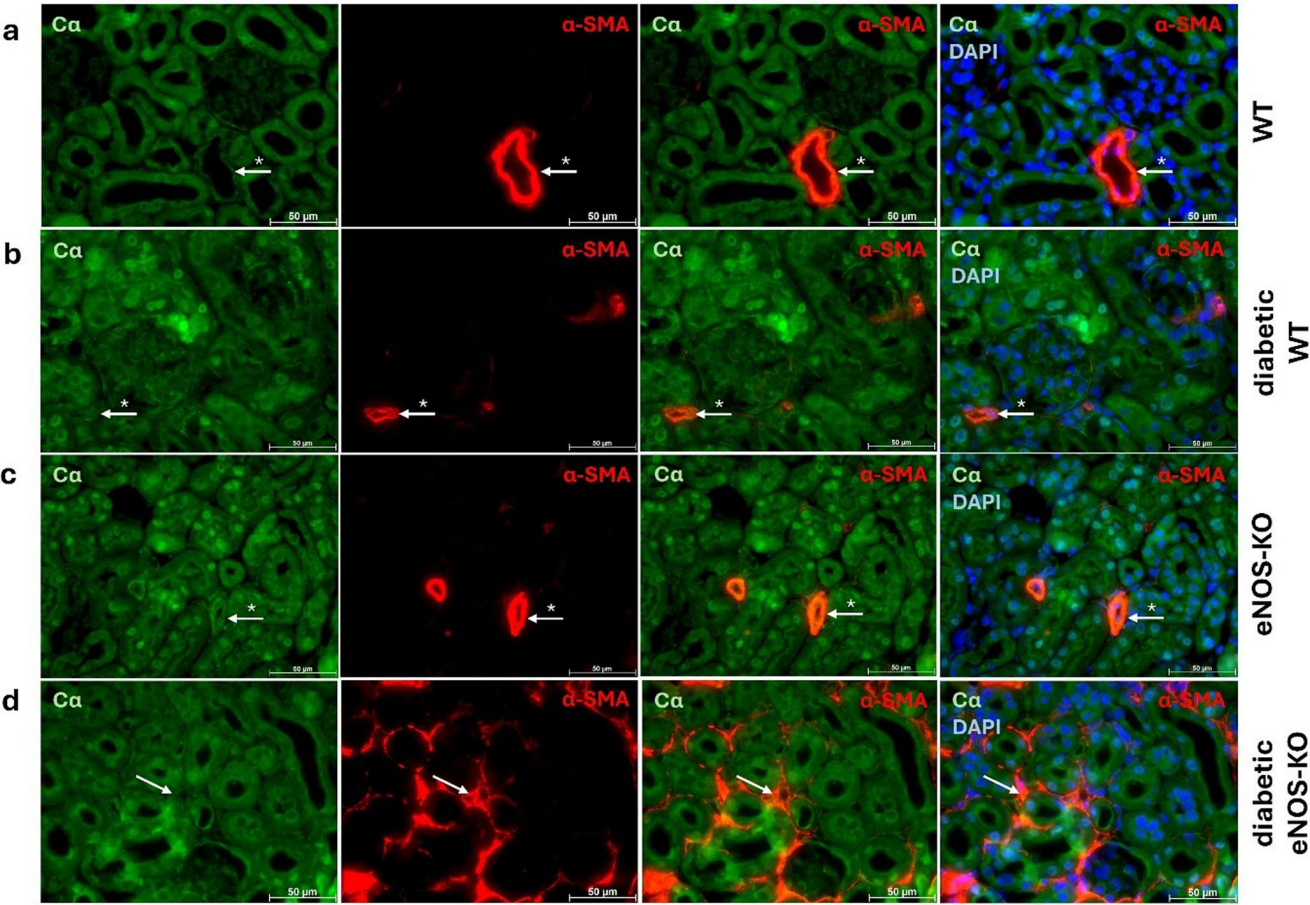
Expression of Cα in vessels and in fibrotic tissue. Myofibroblasts of fibrotic tissue are stained by α-smooth-muscle actin (α-SMA) (red). Expression of Cα (green) is detectable in vessels (**a**-**c**) (indicated by arrow with asterisk) and in myofibroblasts of diabetic eNOS-KO animals (**d**) (indicated by arrow). **a**-**d** Nuclei are stained with DAPI (blue). Scale bars are 50 μm

**Fig. 10:**
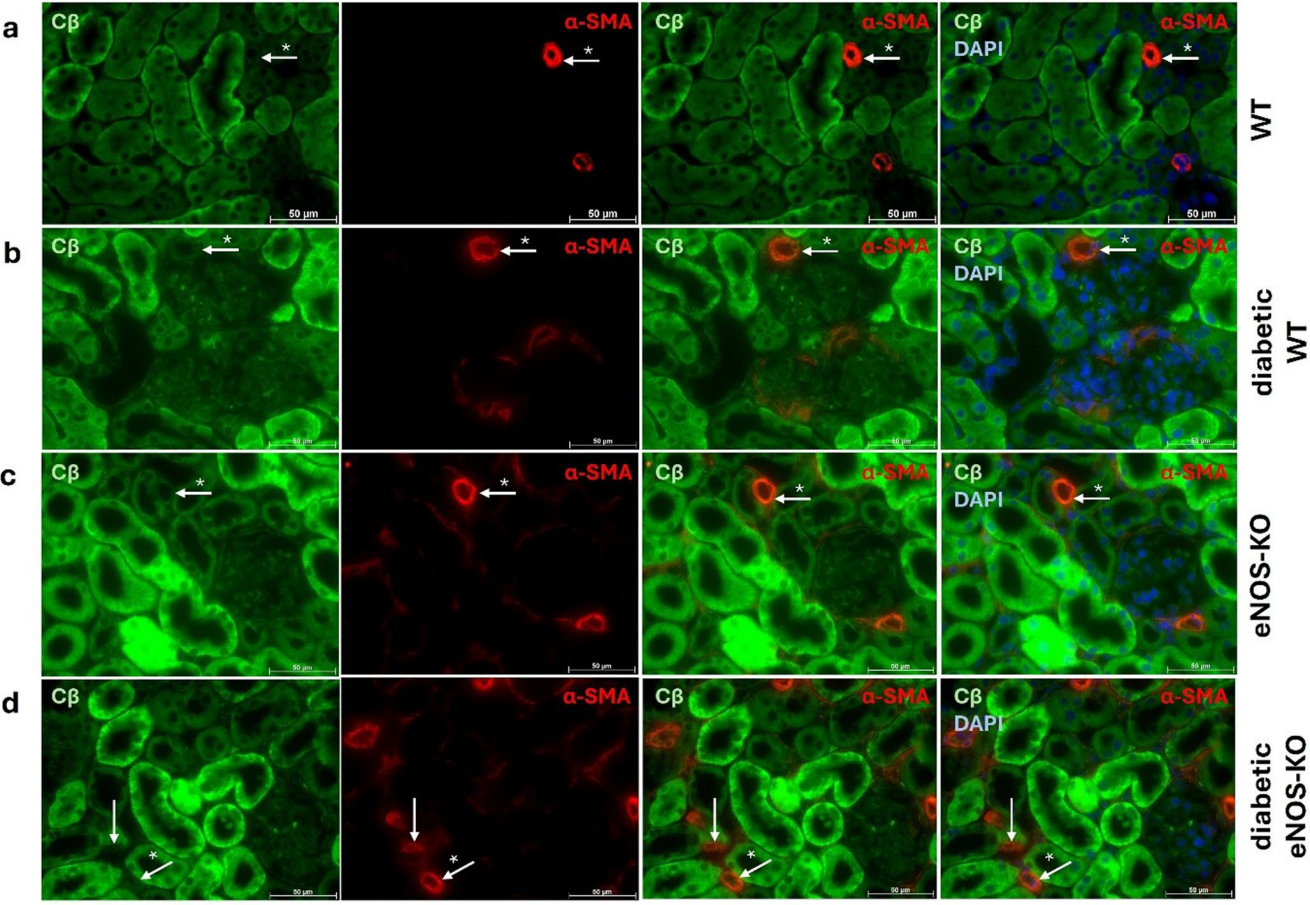
Expression of Cβ in vessels and in fibrotic tissue. Myofibroblasts of fibrotic tissue are stained by α-smooth-muscle actin (α-SMA) (red). Weak expression of Cβ (green) is detectable in vessels (**a**-**d**) (indicated by arrow with asterisk). No expression of Cβ is detectable in myofibroblasts of diabetic eNOS-KO animals (**d**) (indicated by arrow). **a**-**d** Nuclei are stained with DAPI (blue). Scale bars are 50 μm

Kidney fibrosis was analysed by expression of α-smooth muscle actin (α-SMA), a marker for myofibroblast formation and therefore fibrosis. However, it is also expressed in smooth muscle cells like vessels, where it can be used as a marker. Especially in diabetic eNOS-KO mice a high expression of α-SMA is detectable, indicating kidney fibrosis (Figs. 9d and 10d). This finding was previously quantified and reported by our group [13]. We analysed the expression of Cα and Cβ in fibrotic tissue and detected Cα (Fig. 9d) but not Cβ (Fig. 10d) in the myofibroblasts. Further representative images are shown in Online Resource 6d (Cα) and Online Resource 7d (Cβ). Furthermore, Cα was detectable in vessels (Fig. 9a-c, Online Resource 6a-d). Cβ was also detectable in vessels of WT (Fig. 10a, Online Resource 6a), eNOS-KO (Fig. 10c, Online Resource 6c), diabetic WT (Fig. 10b, Online Resource 6b) and diabetic eNOS-KO kidneys (Fig. 10d, Online Resource 6d), but expression was only very weak.

**Fig. 11:**
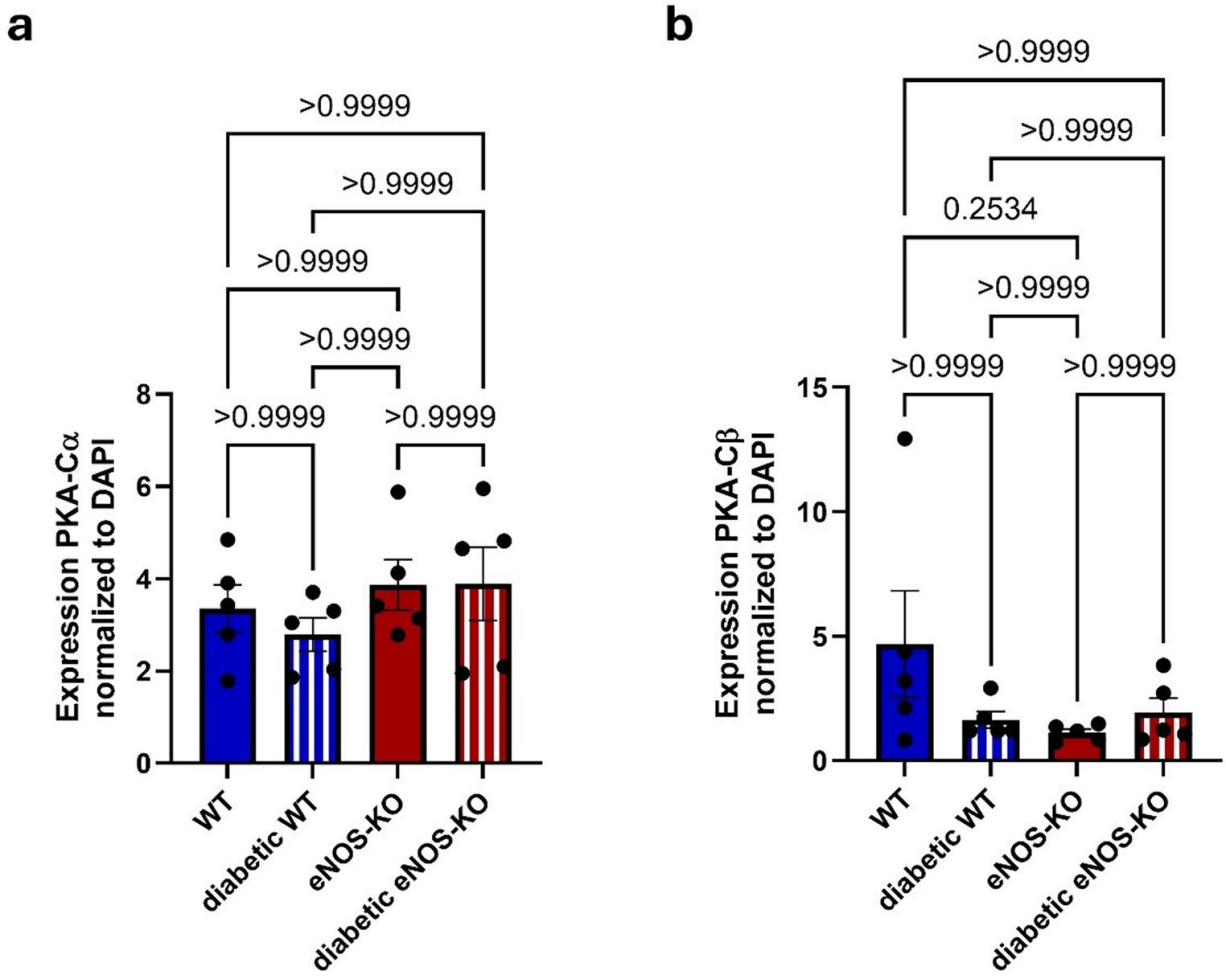
Quantification of Cα (**a**) and Cβ (**b**) expression in the renal cortex of diabetic and non-diabetic wildtype (WT) and eNOS-KO animals. Immunohistochemistry of Cα and Cβ was quantified, and amount of expression was compared between the groups. No significant expression of Cα (**a**) and Cβ (**b**) was detected between the analysed groups. 5 animals per group were used. Expression of Cα and Cβ was normalized to DAPI expression. Data were tested for normality using the Shapiro-Wilk test. **a** Statistical analysis was performed using one-way ANOVA followed by Bonferroni’s post hoc test. Bonferroni-adjusted *p*-values are shown in the graph. **b** Statistical analysis was performed using the Kruskal Wallis test followed by Dunn’s post hoc test. Dunn’s -adjusted *p*-values are shown in the graph

The amount of Cα and Cβ expression was quantified in kidneys of WT, diabetic WT, eNOS-KO and diabetic eNOS-KO mice. However, no significant changes in Cα (Fig. 11a) or Cβ (Fig. 11b) expression were detectable among the analysed groups.

## Discussion

Mammalian cells possess four PKA regulatory subunit isoforms (RIα, RIβ, RIIα and RIIβ) and three catalytic subunit isoforms (Cα, Cβ and Cγ). The aim of our study was to provide a comprehensive overview of differential expression of PKA isoforms in the kidney with a focus on the widely expressed catalytic subunits Cα and Cβ. Especially in the cortex, a high amount of Cα expression was detectable, whereas expression levels in the medulla were lower compared to the cortex. Nonetheless, in both the medulla and the cortex, Cα is detectable in every cell type, but with notable differences in their intracellular localization. As numerous important transport activities and (patho-)physiological processes takes place in the renal cortex, we focused on analysing the expression of the catalytic subunit isoforms in the cortex. Proximal tubules appear to constitutively express the Cα isoform in their nuclei, whereas we could not detect constitutively high levels of Cα in the nuclei of other renal cells like glomerular cells, distal tubules, Loop of Henle, and collecting ducts, where Cα expression was restricted to the cytoplasm. Hence, the difference in subcellular localization indicates distinct, cell-type specific roles of Cα within the kidney. In the nucleus of proximal tubules, Cα may contribute to sustained regulation of gene transcription. However, the mechanism by which Cα is transported to the nuclei remains unclear and a topic of investigation. A possible explanation is the transport through C-kinase anchoring proteins (CKAPs). CKAPs specifically target catalytic subunits to their location site like nuclei, whereas AKAPs rather bind the regulatory subunits to transport PKA to its site of action [17]. However, which isoforms of CKAPs are responsible to translocate Cα to the nuclei, needs to be investigated in individual cell types. Furthermore, in cells that do not show any expression of Cα in the nucleus, it can not be excluded that Cα is translocated to the nuclei under certain physiological or pathological conditions or only upon stimulation. Cβ was detectable in every cell type of healthy murine kidneys, however most of the cells only revealed a very weak expression. The exception are proximal tubules, where we observed high expression levels especially at the basolateral membrane. We did not detect Cβ expression in the nuclei of any of the analysed cell types, indicating that Cβ is either not regulating gene expression or is only translocated to the nuclei under certain circumstances or upon stimulation.

We further analysed which PKA regulatory subunits might interact as a tetrameric holoenzyme with the catalytic subunits. In glomeruli, we found RIα, RIIα and RIIβ expression in the cytosol, whereas RIβ expression was observed in the nuclei of glomerular cells. As Cα and Cβ were found in the cytosol of every glomerular cell, it is possible that Cα or Cβ are building a tetrameric holoenzyme with RIα, RIIα or RIIβ. However, this interaction needs to be investigated for the individual glomerular cell types. In tubular cells, we predominantly found RIβ expression in the nuclei as well as in the cytosol, indicating that RIβ is an interaction partner of Cα or Cβ.

The glomerular filtration barrier (GFB) consists of glomerular endothelial cells (GECs), the glomerular basement membrane, and podocytes [18, 19]. We detected expression of Cα and Cβ in GECs and podocytes, where both isoforms were localized to the cytosol. As cAMP/PKA signalling is involved in the regulation of endothelial function and podocyte integrity [20–23], both catalytic subunits may contribute to maintaining glomerular function. Dysregulation of this pathway has been implicated in diabetic kidney disease, where impaired PKA signalling can promote podocyte injury and apoptosis [24, 25]. We further detected expression of Cα and Cβ in glomerular mesangial cells, which play an important role in maintaining the glomerular structure [26]. While expression of the Cα isoform has been previously reported in rat mesangial cells, the function of the Cβ isoform has not been specifically investigated in mesangial cells until now. Activation of PKA in mesangial cells by cytokines like TGF-β, which is upregulated in diabetic kidneys [27], regulates the transcription of genes involved in the development of fibrosis like fibronectin. This process appears to be mediated by translocation of the Cα isoform to the nucleus and stimulation of CREB-phosphorylation upon TGF-β stimulation [28]. In unstimulated rat mesangial cells, Cα is not detectable in the nucleus [28], which aligns with our findings in healthy murine glomerular cells. These observations suggest that Cα might be an interesting new target for preventing the cytokine induced mesangial fibrosis. Selective inhibitors of Cα activity, like BLU2864, have been already investigated for treatment of polycystic kidney disease [4]. High glucose levels seem to activate PKA signalling in mesangial cells and drive fibrosis [29]. Hence, selective Cα inhibitors might also be of interest for treating kidney diseases like diabetes-induced glomerular fibrosis. Interestingly, in our model of DN, we detect Cα, but no Cβ expression in fibrotic tissue, further supporting the Cα isoform as a potential target. Although stimulation of the cAMP/PKA signalling pathway is known to be antifibrotic [29], it is not clear whether global activation of this pathway is beneficial or whether selective inhibition of Cα activity may be advantageous. Furthermore, high glucose levels seem to regulate PKA signalling differently among the glomerular cell types, which makes it more complicated considering PKA as a target for treatment of kidney diseases. Hence, it would be extremely important to specifically address the different cell types with either an inhibitor or activator when targeting the PKA signalling pathway. For this purpose, nanoparticles-based drug delivery systems that selectively target specific kidney cells might function as a carrier for different drugs. Nanoparticles designed to specifically address mesangial cells have been already investigated in several studies [30–32] and may offer a viable approach for precise modulation of PKA signalling in kidney disease.

Quantification of Cα and Cβ in our diabetic mouse model revealed no significant differences in Cα or Cβ expression compared to non-diabetic animals. However, the relatively small sample size (*n* = 5 animals per group) may have limited the statistical power to detect changes in expression. Importantly, expression levels do not necessarily reflect kinase activity. Therefore, while our data do not indicate major alterations in Cα or Cβ abundance, they do not exclude changes in PKA activity. Further studies assessing isoform-specific PKA activity and downstream signalling pathways will be required to determine whether PKA function is altered in diabetic kidney disease.

Interestingly, we observe high expression of both Cα and Cβ in renal proximal tubule cells (RPTs), but with a different intracellular localization. Cα is expressed all over the cell, whereas the Cβ isoform is strongly expressed especially at the basolateral membrane. Furthermore, Cα seems to be permanently localized in the nuclei of these cells, what is not seen for the Cβ isoform. The different localization of the isoforms suggests diverse functions in regulation of various cellular processes and supports the hypothesis that Cα and Cβ have non-redundant regulatory functions in proximal tubules, which has been already proposed for other cell types like collecting duct cells [33]. In the liver, an important task of PKA is the regulation of gluconeogenesis. Under fasting conditions, transcription and expression of the enzymes needed for the conversion of pyruvate back to glucose are regulated by PKA, which modulates gene transcription by binding to CREBs [34, 35]. Gluconeogenesis also occurs in renal proximal tubules [36], and one possible task of the catalytic subunits might be the regulation of gluconeogenesis by modulating gene transcription of enzymes like PEPCK/PCK1. As we found the Cα subunit to be permanently localized in the nuclei of RPTs, the hypothesis is that this process is regulated by Cα. However, the possible role of PKA in renal gluconeogenesis or which isoform might be involved in its regulation needs to be investigated in more detail. RPTs are highly active metabolic cells. Due to the huge amount of reabsorption of electrolytes, glucose, small proteins or amino acids, RPTs have a high energy demand to facilitate these transport processes. Hence, RPTs require an extremely high mitochondrial density [37, 38]. Multiple studies described the role of PKA in mitochondrial physiology. PKA is tethered to the outer mitochondrial membrane by AKAPs. Inside mitochondria, the mitochondrial PKA targets several substrates like complex I, complex IV or complex V, thereby mediating ATP generation by oxidative phosphorylation. Furthermore, several pro-apoptotic proteins are substrates of PKA, indicating the relevance of PKA signalling in apoptosis. Besides that, mitochondrial proteins, which are important for mitochondrial fusion and autophagy, are targets of PKA [39]. To which extent the catalytic subunits Cα and Cβ regulate mitochondrial function is not fully investigated until now. In the retina, the Cβ isoform is primarily localized to mitochondria, whereas Cα is excluded from this organelle, suggesting Cβ to be important for the regulation of mitochondrial function [40]. However, the mitochondrial expression of Cα and Cβ and their isoform-specific functions in RPT cells have not yet been investigated. Addressing these questions will require further studies using higher resolution images and functional analyses.

Other parts of the tubular system, including the loop of Henle, distal tubules, and collecting ducts, also expressed Cα and Cβ, although Cβ expression was markedly weaker. PKA signalling has been implicated in several functions within these nephron segments, including NKCC2 trafficking in the thick ascending limb and AQP2 trafficking in collecting ducts [41–47]. While the specific catalytic isoform involved remains largely unknown, our data demonstrate the presence of Cα and Cβ in these structures, suggesting their functional involvement. Previous phosphoproteomic analyses in collecting duct cells lacking either Cα or Cβ indicated non-redundant functions of the two isoforms [33]. As AQP2 is markedly decreased in Cα null cells, the Cα isoform is suggested to be the predominant catalytic isoform for the regulation of AQP2 [48]. Given the central role of cAMP/PKA signalling in collecting duct function and its involvement in nephrogenic diabetes insipidus, modulation of this pathway remains of therapeutic interest [49]. Furthermore, PKA signalling via Cα or Cβ may contribute to the regulation of transport processes and renal autoregulation through NKCC2, which is expressed in the macula densa [50]. We also detected strong Cα but only weak Cβ expression in juxtaglomerular cells, suggesting a predominant role of Cα in renin regulation. Since cAMP/PKA signalling controls renin expression and also influences the transition of renin cells toward profibrotic phenotypes, the pathway could represent a potential target for interventions to prevent kidney damage and fibrosis [51–53].

**Fig. 12:**
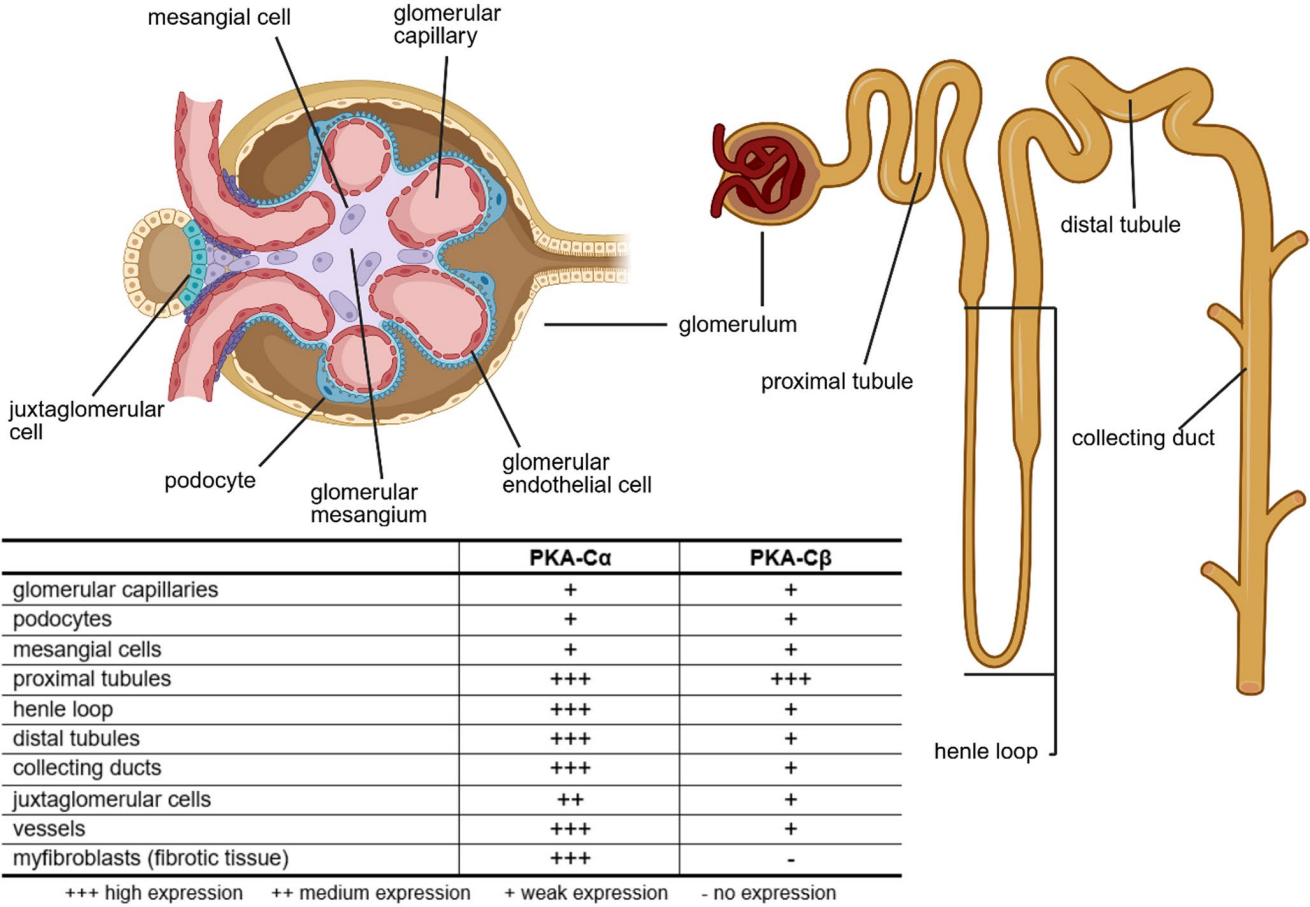
Summary of Cα and Cβ expression. Overview about nephron structure and expression of Cα and Cβ in the different kidney segments. Created in BioRender. Fuchs, S. (2026) https://BioRender.com/vau3hox

In summary, with this work we provide an overview on the expression and localization of the PKA catalytic subunits Cα and Cβ in the kidney (Fig. 12). Interestingly, the proximal tubules reveal a high expression of both isoforms with differences in their intracellular localization. Furthermore, only the proximal tubules express Cα in their nuclei, whereas Cα expression is only cytoplasmatic in all other renal cell types. The differential expression pattern of Cα and Cβ in most of the renal cell types suggests non-redundant regulatory functions of the different isoforms. As PKA signalling is not only important in healthy, but also in diseased kidneys, the distinct localization of these isoforms might provide a basis not only for investigation of their physiological renal functions but also for a targeted therapy.

## Supplementary Information

Below is the link to the electronic supplementary material.

Supplementary Material 1.

## Data Availability

The generated datasets are available from the corresponding author upon reasonable request.
