# Supplementary material for: Expression of protein kinase A catalytic subunits in healthy and diseased mouse kidneys
Source: Pflugers Arch. 2026 Jun 26;478(7):60. doi: 10.1007/s00424-026-03190-z (PMC13303448; doi:10.1007/s00424-026-03190-z)
Supplement: Supplementary file 1 — Supplementary Material 1. [file 424_2026_3190_MOESM1_ESM.docx]

**Supplementary Information (SI)**

For the article

“Expression of protein kinase A catalytic subunits in healthy and diseased mouse kidneys”

Authors:

Sally Fuchs^1^, Michael Majer^1^, Yuliang Ma^2^, Manuela Harloff^1^, Susan Taylor^2^ and Jens Schlossmann^1^

^1^ Department of Pharmacology, University of Regensburg, Regensburg, Germany

^2^ Department of Pharmacology, University of California, San Diego, United States of America

Corresponding Authors:

Sally Fuchs and Jens Schlossmann

Department of Pharmacology, University of Regensburg, Regensburg, Germany

E-Mail: [sally.fuchs@chemie.uni-regensburg.de](mailto:sally.fuchs@chemie.uni-regensburg.de); jens.schlossmann@chemie.uni-regensburg.de

Submitted in Pflügers Archiv - European Journal of Physiology


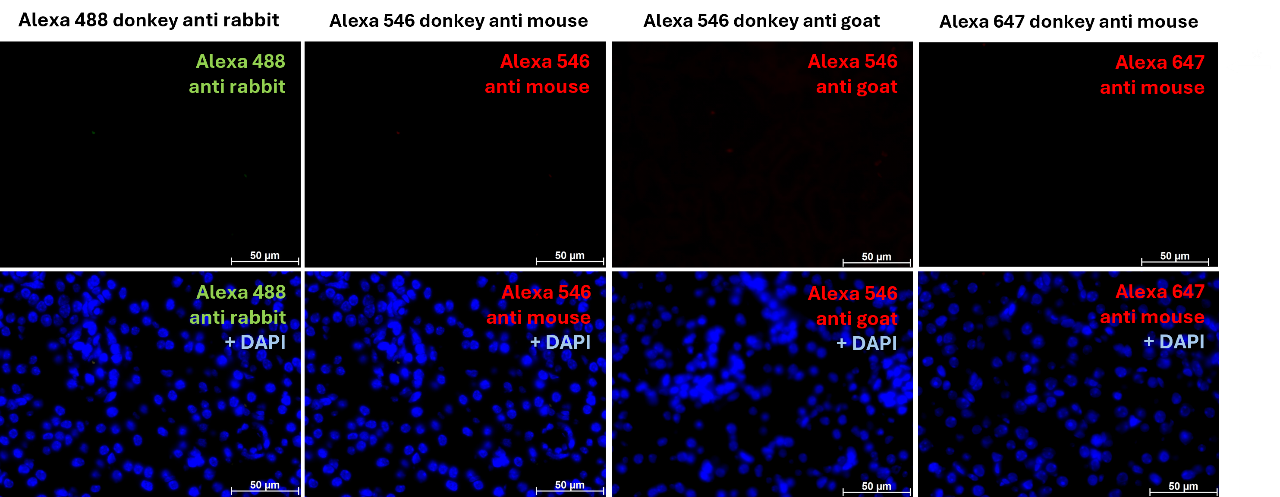


Online Resource 1 Antibody control for secondary antibodies used in Figs. 2a, 2b and Figs. 3 to 10

Kidney sections that were used as negative controls for corresponding IHC data showed no detectable signal after incubation with the secondary antibodies Alexa 488 donkey anti rabbit, Alexa 546 donkey anti mouse, Alexa 546 donkey anti goat and Alexa 647 donkey anti mouse. Scale bars are 50 µm. Nuclei are stained in blue (DAPI)


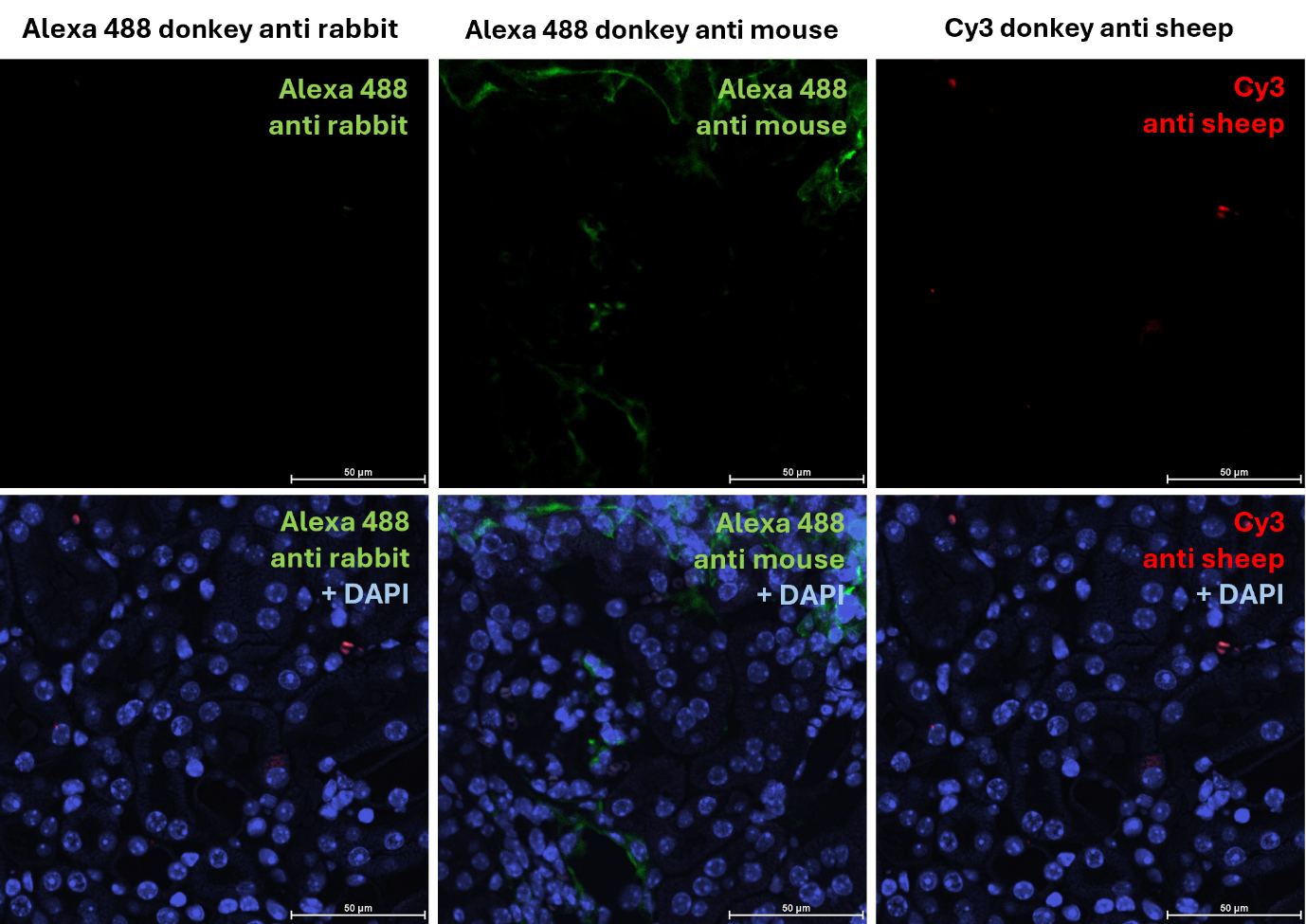


Online Resource 2 Antibody control for secondary antibodies used in Fig. 2c

Kidney sections that were used as negative controls for corresponding IHC data showed no signal after incubation with the secondary antibodies Alexa 488 donkey anti-rabbit and Cy3 donkey anti sheep. Alexa 488 donkey anti mouse showed an unspecific signal in the tubular system of the renal cortex. Alexa 488 donkey anti mouse is used for detection of RIα, RIIα and RIIβ (Figure 2C), which are mainly localized to the glomerulus. In the glomeruli no unspecific signal of Alexa 488 donkey anti mouse was detectable. Scale bars are 50 µm. Nuclei are stained in blue (DAPI)


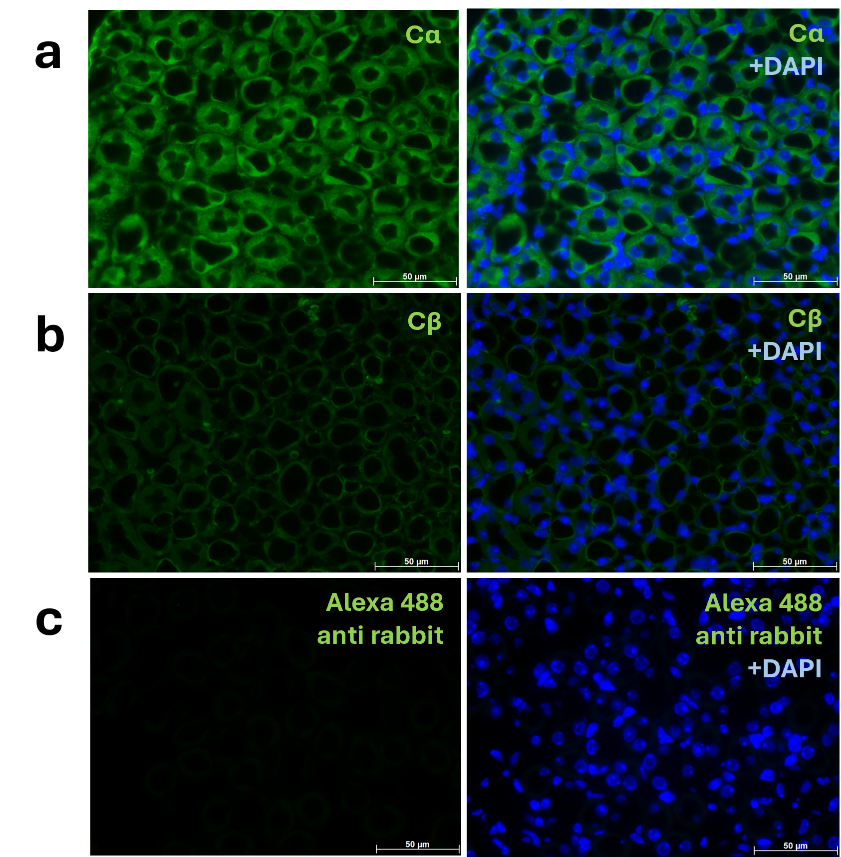


Online Resource 3 Expression of Cα (a) and Cβ (b) in the medulla

a Expression of Cα is detectable in the renal medulla. b Only very week expression of Cβ can be found in the medulla. c Kidney sections used as negative controls showed no signal after incubation with the used secondary antibody Alexa 488 donkey anti rabbit in the medulla. Scale bars are 50 µm. Nuclei are stained in blue (DAPI)


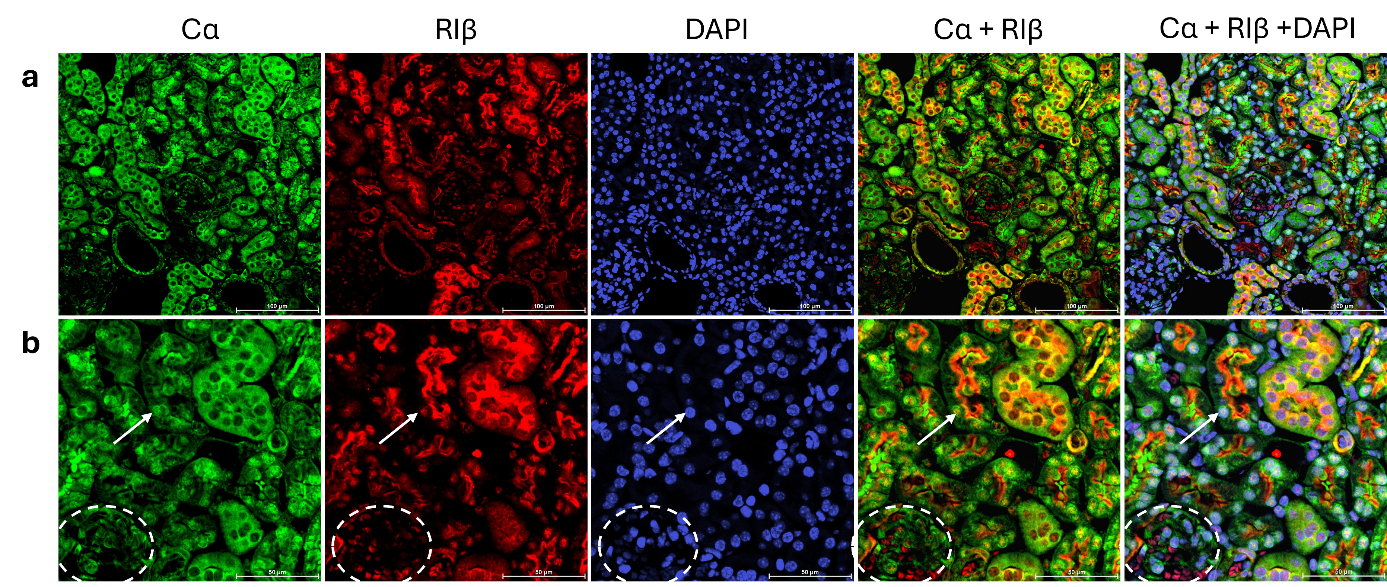


Online Resource 4 Colocalization analysis of Cα and RIβ

a Scale bars are 100 µm. b Scale bars are 50 µm. Glomeruli are visualized by white dashed lines. a Cα and RIβ are co-expressed in several cell types in the renal cortex. b Confocal images with higher magnification show that Cα and RIβ are co-expressed – among other cells – in proximal tubules (indicated by arrows). Proximal tubules were identified as the only cell type expressing Cα in their nuclei. RIβ is also expressed in the nuclei of proximal tubules and moreover, is strongly expressed at the apical membrane of the proximal tubules. In other cell types, RIβ is expressed over the whole cell body. In glomeruli, RIβ expression can be detected especially in nuclei. Nuclei are stained in blue (DAPI)


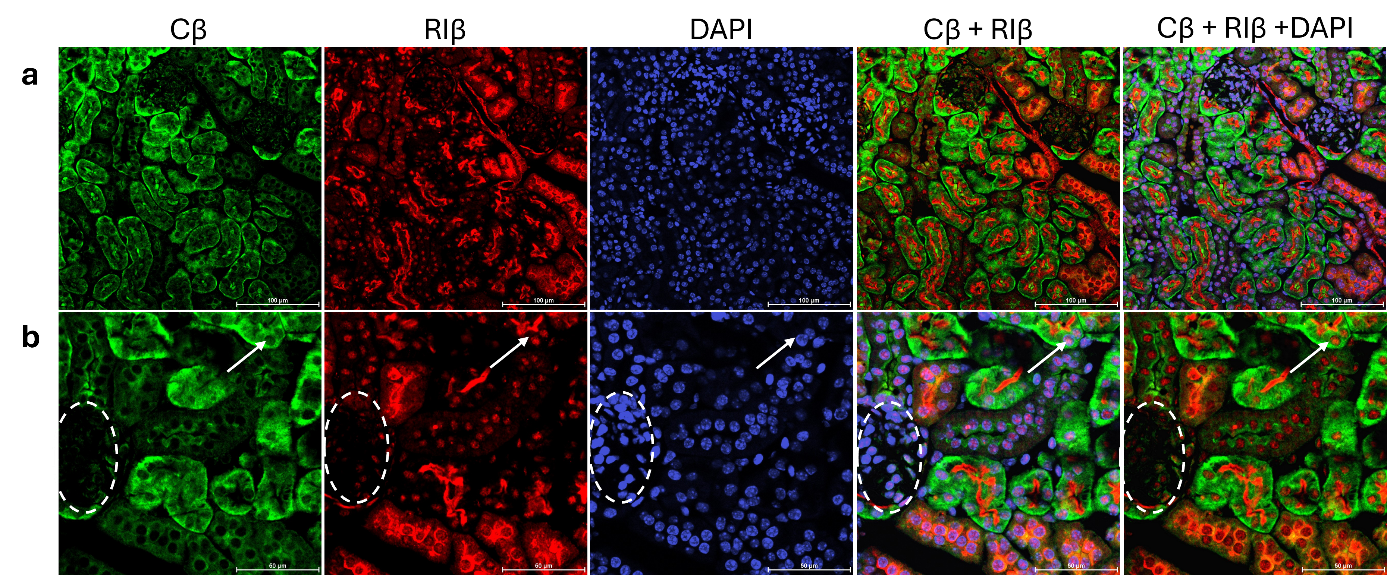


Online Resource 5 Colocalization analysis of Cβ and RIβ

a Scale bars are 100 µm. b Scale bars are 50 µm. Glomeruli are visualized by white dashed lines. a Cβ and RIβ are co-expressed in several cell types of the renal cortex. b Confocal images with higher magnification show that Cβ and RIβ are co-expressed – among other cells – in proximal tubules (indicated by arrows). Proximal tubules were identified as they express high levels of Cβ compared to other cell types in the renal cortex. RIβ is expressed in the nuclei of the proximal tubules and furthermore, is strongly expressed at the apical membrane of the proximal tubules. In other cell types, RIβ is expressed over the whole cell body. In glomeruli, RIβ expression can be detected especially in nuclei. Nuclei are stained in blue (DAPI)


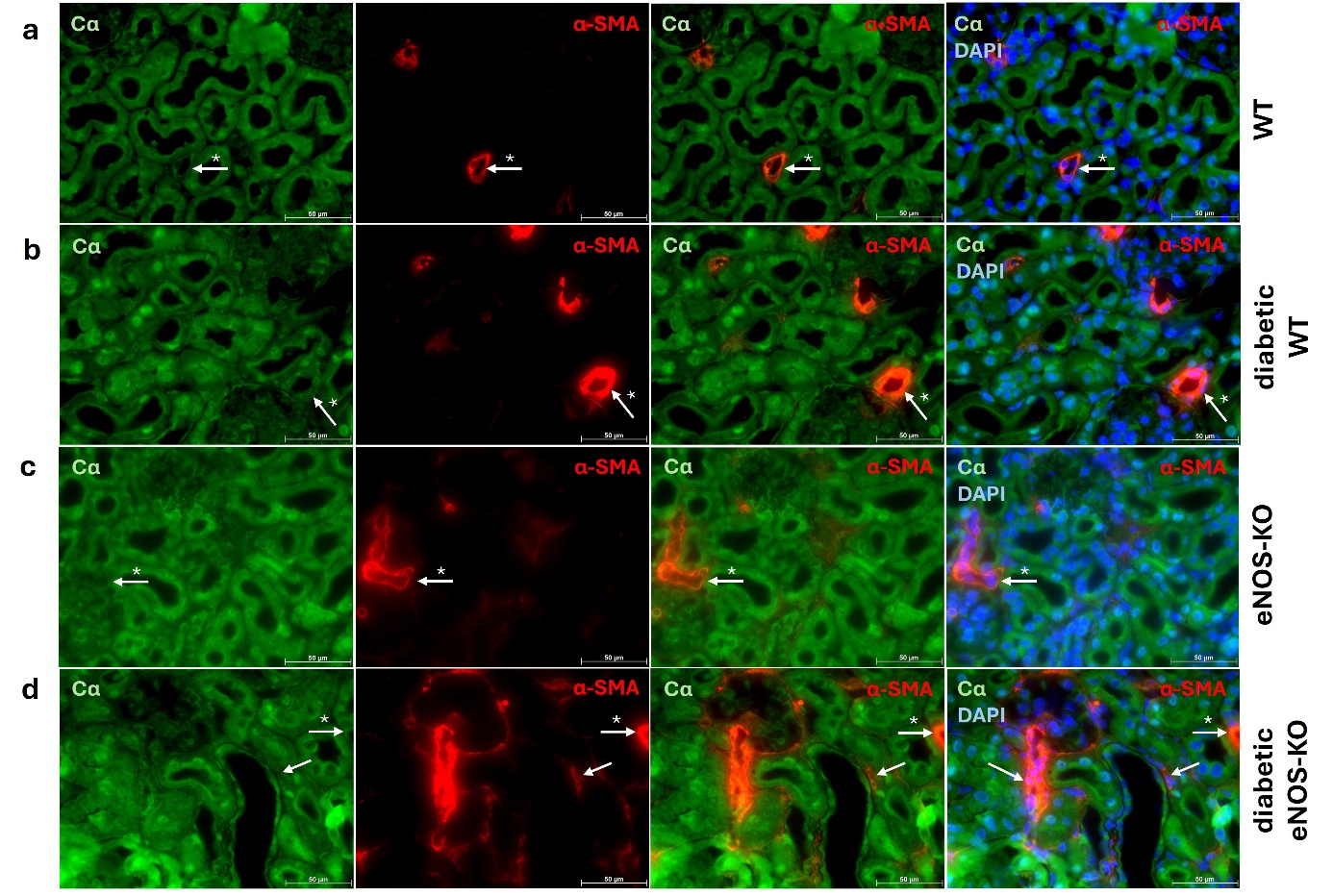


Online Resource 6 Expression of Cα in vessels and in fibrotic tissue

Myofibroblasts of fibrotic tissue are stained by α-smooth-muscle actin (α-SMA) (red). Expression of Cα (green) is detectable in vessels (a-d) (indicated by arrow with asterisk) and in myofibroblasts of diabetic eNOS-KO animals (d) (indicated by arrow). a-d Nuclei are stained with DAPI (blue). Scale bars are 50 µm


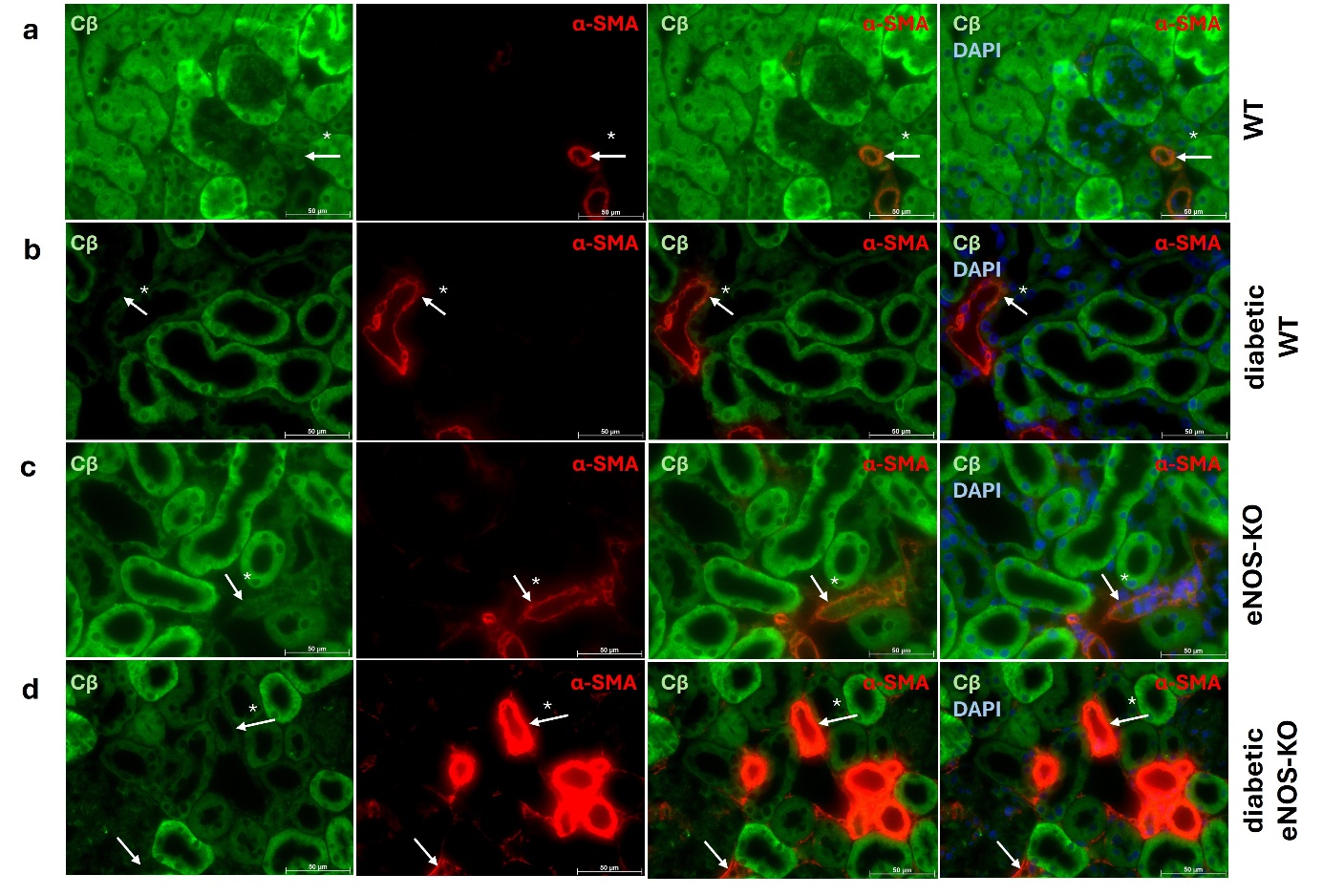


Online Resource 7 Expression of Cβ in vessels and in fibrotic tissue

Myofibroblasts of fibrotic tissue are stained by α-smooth-muscle actin (α-SMA) (red). Weak expression of Cβ (green) is detectable in vessels (a-d) (indicated by arrow with asterisk). No expression of Cβ is detectable in myofibroblasts of diabetic eNOS-KO animals (d) (indicated by arrow). a-d Nuclei are stained with DAPI (blue). Scale bars are 50 µm
